# Biokinetics and Internal Dosimetry of Tritiated Steel Particles

**DOI:** 10.3390/toxics10100602

**Published:** 2022-10-12

**Authors:** Rachel Smith, Michele Ellender, Chang Guo, Derek Hammond, Adam Laycock, Martin O. Leonard, Matthew Wright, Michael Davidson, Véronique Malard, Mickaël Payet, Christian Grisolia, Eric Blanchardon

**Affiliations:** 1 Radiation Chemicals and Environmental Hazards, UK Health Security Agency, Harwell Campus, Didcot OX11 0RQ, UK; 2 Biosciences and Biotechnology Institute of Aix-Marseille (BIAM) (Aix-Marseille University, French Alternative Energies and Atomic Energy Commission (CEA), French National Centre for Scientific Research (CNRS)), 13108 Saint Paul-Lez-Durance, France; 3 Institute for Magnetic Fusion Research (IRFM), French Alternative Energies and Atomic Energy Commission (CEA), 13108 Saint-Paul-lez-Durance, France; 4 Institut de Radioprotection et de Sûreté Nucléaire (IRSN), 92260 Fontenay-aux-Roses, France

**Keywords:** tritium, tritiated steel particles, dissolution, rat, biokinetics, dosimetry, dose coefficients

## Abstract

Decommissioning fission and fusion facilities can result in the production of airborne particles containing tritium that could inadvertently be inhaled by workers directly involved in the operations, and potentially others, resulting in internal exposures to tritium. Of particular interest in this context, given the potentially large masses of material involved, is tritiated steel. The International Commission on Radiological Protection (ICRP) has recommended committed effective dose coefficients for inhalation of some tritiated materials, but not specifically for tritiated steel. The lack of a dose coefficient for tritiated steel is a concern given the potential importance of the material. To address this knowledge gap, a “dissolution” study, in vivo biokinetic study in a rodent model (1 MBq intratracheal instillation, 3-month follow-up) and associated state-of-the-art modelling were undertaken to derive dose coefficients for model tritiated steel particles. A committed effective dose coefficient for the inhalation of 3.3 × 10^−12^ Sv Bq^−1^ was evaluated for the particles, reflecting an activity median aerodynamic diameter (AMAD) of 13.3 µm, with the value for a reference AMAD for workers (5 µm) of 5.6 × 10^−12^ Sv Bq^−1^ that may be applied to occupational inhalation exposure to tritiated steel particles.

## 1. Introduction

The decommissioning of fission and fusion facilities can result in the production of airborne particles containing tritium that could inadvertently be inhaled by workers directly involved in the operations, and potentially others, resulting in internal exposures to tritium. Of particular interest in this respect, given the potentially large masses of material involved, are tritiated concrete and steel. Tritium is produced in nuclear reactors mainly by the ternary fission of the uranium fuel and neutron activation of lithium and boron, with quantities dependent on factors including reactor design [1]. Tritium is then transported by primary fluids and diffusion through reactor walls and circuits. It may thus contaminate both steel and concrete. As an example, concrete and metal waste from the decommissioning of French nuclear power plants contain a few Bq to hundreds of Bq of tritium per gramme [2]. The use of tritium in fusion facilities will also result in the generation of tritiated wastes expected to be dominated by structural materials, including steels [3,4].

Austenitic stainless steels are of particular interest as they have been used extensively as a construction material for nuclear reactors, and the solubility and diffusivity of hydrogen and its isotopes through austenitic stainless steels is relatively high [5,6]. The exposure of metal surfaces to tritium gas results in an exchange with hydrogen in surface-adsorbed water. Tritium can pass from this surface into the metal via bulk diffusion and grain boundary diffusion across the surface oxide layer [7]. Steel particles containing tritium may therefore be generated in the decommissioning process by the cutting of pipes or structural components made of steel. For example, reciprocating saws would likely be used for cutting components made from thin steel in order to reduce heat and subsequent tritium release [8].

Workers undertaking decommissioning activities involving tritiated materials would be expected to wear appropriate personal protective equipment to limit exposures; however, it is possible that airborne tritiated particles produced during such activities may inadvertently be inhaled by workers as a result of accidents or incidents, including the use of inappropriate or damaged respiratory protection. Any inhaled particles will deposit in the extrathoracic airways and the lungs. Some of the inhaled particles will be cleared to the gut, while the rest remain on the airway walls, especially in the deep lung. Tritium will be released from the particles and absorbed in blood, quickly mixing with the whole pool of body water before being cleared in urine and by other routes of excretion. While the radionuclide is present in the body, the short-range beta radiation emitted by tritium delivers a dose to all body organs, but primarily the lung. Importantly, the rate of release of tritium from particles drives the distribution of this dose between the lung and other organs.

As it is an indicator of radiological detriment, the radiation doses incurred by workers must be evaluated, kept as low as reasonably achievable, and, in any case, below regulatory limits. Inhalation dose coefficients applicable to the tritiated particles generated during decommissioning operations are important in estimating these radiation doses that will form an input to decisions on appropriate dose and risk management procedures and processes. The International Commission on Radiological Protection (ICRP) have recommended committed effective dose coefficients for the inhalation of some tritiated materials [9]. For example, for carbon tritide and hafnium tritide, a value of 2.6 × 10^−10^ Sv Bq^−1^ is recommended, whereas for tritium tritide, zirconium tritide, tritiated glass fragments and luminous paint, a value an order of magnitude lower of 2.4 × 10^−11^ Sv Bq^−1^ is recommended. Both of these values are greater than the effective dose coefficient for tritiated water (HTO), 2.0 × 10^−11^ Sv Bq^−1^. The ICRP have not recommended a specific inhalation dose coefficient for tritiated steel particles, but have indicated that for all unspecified particulate tritiated materials, a value of 2.4 × 10^−11^ Sv Bq^−1^ is recommended. The lack of a specific dose coefficient for tritiated steel is a concern given the potential importance of the material. To address this knowledge gap, experimental studies using model tritiated steel particles and associated modelling studies have been undertaken to support the development of appropriate biokinetic models allowing the estimation of relevant dose coefficients for inhalation.

The ICRP-recommended dose coefficients for tritiated materials were derived primarily from experimental studies in animals, which have shown significant differences in the biokinetics and, in particular, the rate of release of tritium from different types of tritiated particles in the lung. This release is frequently referred to in the literature as “dissolution”, but although some limited dissolution of particles may occur, resulting in the release of tritium and also the transformation of tritium gas into HTO, the primary process of interest is the release of tritium from particles into biological tissues in the absence of significant dissolution of the particle matrix. To investigate the rate of release of tritium from tritiated steel particles (via both processes), an in vitro tritium release study was undertaken. In line with previous studies of tritiated metallic particles [10,11,12], an in vivo study in a rodent model was also undertaken to determine the biokinetics of the tritium associated with the tritiated steel particles. This generated data on the tritium content of organs and tissues and excreta samples over time, which were used to develop a biokinetic model for the rat and also to generate relevant biokinetic parameters for use in a human model to determine specific dose coefficients.

## 2. Materials and Methods

### 2.1. Tritiated Steel Particles

Tritiated steel particles were provided by CEA Saclay (French Alternative Energies and Atomic Energy Commission) and details of their production and characterisation have previously been described [13,14]. In brief, they were produced (see details below) by tritiating commercially available (austenitic) 316L steel powder (Goodfellow Cambridge Limited, Huntingdon, UK) with a nominal particle diameter of 3 µm. Scanning electron microscopy images indicated that the particles are spherical in shape, with diameters from <1 µm to 8 µm. The particle size distributions of the steel powder were determined using an automated static optical microscopy imaging system, Morphologi 3 (Malvern Panalytical Ltd., Malvern, UK) with an operational size range of 0.5–1000 µm. Fitting the volume-based primary particle size distribution to a lognormal function produced a volume median diameter of 4.7 µm with a geometric standard deviation of 1.35. Because of the relatively high density of steel, this is equivalent to an aerodynamic median diameter of 13.3 µm (geometric standard deviation, 1.35) [13]. The tritiation process included two steps of reduction at 450 °C under H_2_ at high pressure for 2 h, followed by exposure to tritium gas at 450 °C for 2 h and a degassing phase to eliminate unbound tritium to produce particles with a specific activity of 1 MBq/mg [14], confirmed by liquid scintillation counting following particle digestion.

### 2.2. In Vitro Tritium Release Study

The purpose of the in vitro tritium release study was to explore the bioavailability of the tritium present in the tritiated steel particles. This was undertaken by determining the release of tritium from the particles in a physiologically relevant, simulated biological fluid. There are a range of simulated biological fluids used in pulmonary-relevant studies [15]. The main lung-associated simulated biological fluids are of three types: lung lining fluid models, lung interstitial fluid models and lysosomal fluid models. The first represent the fluid that inhaled materials make initial contact with and form the largest group of simulated fluids. The alveolar lining fluid is pH 7.4 and contains a range of salts and also a low concentration of phospholipids from the lung surfactant layer. However, the majority of simulants focus on mimicking the pH and overall salt concentration only, as including phospholipids and other minor components is complex and makes them difficult to use in practice, and their inclusion is not anticipated to alter dissolution significantly. For this study, a version of Gamble’s solution [16], a well-known lung lining fluid simulant, was used. However, it is important to reflect that following inhalation, some particles will be removed from the lungs via the mucociliary escalator to the gastrointestinal (GI) tract. The more acidic environment in the first stages of the GI tract (GI tract pH 1.5) would be expected to have a more aggressive effect upon particles, potentially leading to higher tritium release rates and thus bioavailability. Similarly, inhaled particles are also cleared by phagocytosis by alveolar macrophages and other immune cells, resulting in their accumulation in cell lysosomes, which also present an acidic environment. For these reasons, the use of lung lining fluid is expected to give an indication of tritium release from particles, which will be more relevant in the short-term than the longer-term following exposure. All other studies of tritium release from tritiated particles in the literature have used a lung lining fluid simulant, so this approach will also allow direct comparisons with other results in the literature.

A freshly prepared Gamble’s solution [16] (Table S1) was used as a lung lining fluid simulant (LLFS). The pH of the solution was 7.4. A volume of 1.25 mL of LLFS was added to a mass of 0.5 mg of particles (0.5 MBq) and agitated. One mL of the resulting suspension was used for the study. The procedure used was similar to that previously used for in vitro tritium release studies with tritium-bearing carbon particles [17,18]. Dialysis tubing (Sigma-Aldrich, seamless cellulose tubing, 14,000 molecular weight cut-off, 6 mm inflated diameter) was prepared using the following procedure. After cutting to the required length (approximately 10 cm), the dialysis tubing was washed in running de-ionised water for 3–4 h to remove any glycerine present. Sulphur compounds were removed by washing with 2% (*w*/*v*) sodium hydrogen carbonate/1 mM EDTA at 80 °C for 30 min followed by a thorough rinse in de-ionised water. The tubing was then soaked in the LLFS overnight before use. The prepared tubing was sealed at one end and 1 mL of particle suspension (0.4 mg/mL) in LLFS was dispensed into the tubing, which was sealed completely and immersed in 200 mL of LLFS inside a plastic screw-capped jar. The jar was suspended in a water bath at 37 °C, covered and kept in the dark. At pre-determined time intervals (5 min, 30 min, 1 h, 2 h, 4 h, 1 d, 2 d, 3 d, 6 d, 10 d and 14 d), 1 mL samples from the LLFS surrounding the tubing were removed for analysis and the volume replenished with fresh LLFS. The system was agitated prior to and post sampling. The tritium content of the simulant was determined by liquid scintillation counting. To a 1 mL sample, 15 mL scintillation cocktail (Gold Star, Meridian Biotechnologies, Tadworth, UK) was added. The mixture was thoroughly agitated and then kept in the dark to allow the chemiluminescence of the scintillant to subside, before counting on a liquid scintillation counter (Tri-Carb 3110, PerkinElmer, Waltham, MA, USA).

### 2.3. In Vivo Instillation Study

**Figure 1:**
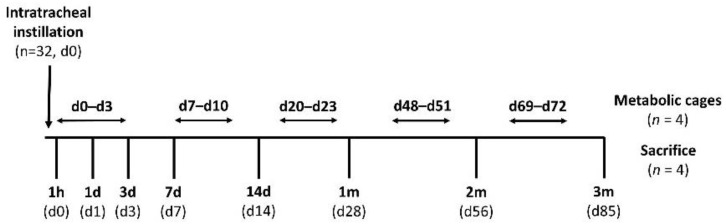
Experimental scheme.

The experiments were performed within the legal framework of the United Kingdom under a Project Licence granted by the Home Office of Her Majesty’s Government. All procedures involving the animals were performed in accordance with the Animals (Scientific Procedures) Act 1986 (Licence number PPL 30/3071, approved on 23 May 2013 by the UK Secretary of State). Thirty-two female, pathogen-free, Sprague Dawley (SD) rats were purchased from Harlan, UK. Following a single instillation, the rats (*n* = 4) were sacrificed at several times post-exposure to 85 days and organs and tissues taken for tritium analysis. For various periods during the study, the animals (*n* = 4) were placed in metabolic cages and excreta collected for analysis. The overall design is presented in Figure 1.

The particles used were provided in eight glass vials, each containing 5 mg of tritiated steel particles of specific activity 1 MBq/mg. The contents of each vial were used for a set of four animals. Just prior to the instillation procedures, 1 mL 0.9% NaCl solution was added to each vial and the lid resealed. The vials were then sonicated for 20 min in an ultrasonic bath (Ultrawave, Cardiff, UK). The sonication step is intended to break up agglomerates; however, after sonication, the suspensions rapidly sediment. The vials were then shaken by hand immediately prior to an aliquot being taken for instillation.

The intratracheal instillation procedure was based on that used in previous studies (e.g., [19]). The animals were anaesthetised using isoflurane (3.5%) and then a marked polythene tube (1.6 mm external diameter, Type PP160, Instech, Philadelphia, PA, USA) passed via the mouth to the first bifurcation of the trachea. The sample vial was swirled and a 0.2 mL (1 MBq) sample drawn into a marked section of smaller diameter polythene tubing (1 mm external diameter, BTPE-50, Instech, Philadelphia, PA, USA) attached to an Eppendorf pipette. The 0.2 mL sample was then discharged into the lungs through the smaller tube placed inside the first one and the Eppendorf pipette used to discharge the liquid. The inner tube was marked at the length of the outer tube to prevent overextension into the lung. After instillation, the suspension was allowed to drain under gravity into the pulmonary region of the lungs as the rat regained consciousness.

At various times post-exposure, the animals were anaesthetised by inhalation of isoflurane (induced at 5%, maintained at 1.5–2% in 100% oxygen) followed with overdosed pentobarbital sodium administration (around 1 mL/rat) intraperitoneally. The abdomen was opened and a small opening cut in the diaphragm near the xiphoid process. The rib cage was removed to expose the lungs and heart. A blood sample was taken from the left ventricle and samples of the other organs (lung, liver, kidney and muscle) collected. Details of samples collected are indicated in Table S2. At various intervals during the study (Figure 1), the animals (*n* = 4) were placed into individual metabolic cages and urine (1 mL) and faecal samples (~20 mg) collected for three 24 h periods. The total mass of faeces produced was determined for each period and a small number of measurements of total daily urine production undertaken.

### 2.4. Biological Sample Analysis

The samples were collected in pre-weighed 25 mL scintillation vials. The samples included solid tissues (lung, liver, kidney and muscle) of approximately 200 mg (range 80–600 mg), 1 mL urine and 20 mg faeces. To digest the biological samples, GoldiSol tissue solubiliser (Meridian Biotechnologies, Tadworth, UK) was added directly to the sample, for solid samples, 1 mL GoldiSol per 100 mg of sample or 2 mL total, whichever was the lower, was added, and for urine samples, 1 mL GoldiSol was added. The closed vials were placed in an oven at 50°C until digestion was complete. The samples were then removed and allowed to cool, stored at −20 °C until ready to be counted, and then 15 mL of the Gold Star scintillation cocktail (Meridian Biotechnologies, Tadworth, UK) was added to the vial and shaken for 10 s. The vials were dark adapted for 1 h prior to counting. The vials were then counted on a liquid scintillation analyser (Tri-Carb 3110, PerkinElmer, Waltham, MA, USA).

The organically bound tritium (OBT) in a number of liver samples was determined using a pyrolysis approach. For the purposes of this analysis, OBT was defined as tritium that is not driven from the sample by heating at a temperature of 180 °C. The weighed samples were dried and then combusted in a Pyrolyser-6 Trio three-zone furnace (Raddec International Ltd., Southampton, UK). The water vapour and other gaseous releases from the heated and combusted sample were collected separately in pre-weighed bubblers containing dilute nitric acid (10 mL of 0.05M HNO_3_). The sample was initially heated with compressed air passing over the sample. The temperature was raised to 180 °C in the sample zone and held for 1 h 30 min to release all HTO, and then the HNO_3_ bubblers were changed before the furnace temperature was increased to 900 °C. Once the temperature was more than 500 °C, a pure O_2_ atmosphere was introduced into the tubes at ≈0.25 L/min. To ensure the complete oxidation of the samples, the temperature was held at 900 °C with O_2_ flowing for at least 30 min. The tritium captured during this second step is defined as OBT. For some samples, a single bubbler was used to determine the total available tritium (see Table S3 for a summary of the operating cycles for the two different approaches). To determine the tritium level in the bubbler solution, 7 mL of the prepared solution was pipetted into a plastic scintillant vial containing 13 mL of Gold Star scintillant and, following cooling and dark adapting as for the digestion method, were counted in the liquid scintillation counter (Tri-Carb 3110, PerkinElmer, Waltham, MA, USA).

### 2.5. Rat Biokinetic Model

The biokinetic data produced by the in vivo study was used to develop a model of the biokinetics of the tritiated particles in the rat. Biokinetic models for radionuclides represent the incorporation of the radionuclides, their time-dependent distribution in organs and tissues and their clearance by excretion. Formally, they are typically represented as compartmental models, i.e., they consist of compartments representing pools of activity (e.g., activity retained in an organ, in a tissue, in a region of an organ/tissue or as a specific chemical form of the radionuclide within an organ/tissue), that are connected by transfer pathways associated with constant transfer rates (in d^−1^). Mathematically, the model translates into a set of first-order differential equations, the solution of which gives the model-predicted retention and excretion of radionuclides. The whole rat biokinetic model was implemented in the SAAM (Simulation Analysis and Modeling) II software (The Epsilon Group, Charlottesville, VA, USA) [20] and the transfer rates between compartments were fitted to the available biokinetic data.

### 2.6. Estimated Dose Coefficients for Workers Exposed to Tritiated Steel Particles

Because of differences in the metabolism between species, the biokinetic model derived from experimental rat data cannot be directly extrapolated to humans. Therefore, the dose calculations for workers were based on the following ICRP models: the human respiratory tract model (HRTM) [21], the human alimentary tract model (HATM) [22] and the systemic model for HTO [9]. However, absorption parameters (defining the rate of release of tritium from steel particles in the lung) are assumed to be independent of species [9] and therefore the values evaluated for tritiated steel particles in rats were assumed to apply to human workers as well, and were used here in combination with ICRP models to propose material-specific dose coefficients for tritiated steel particles. The dose calculations for workers were performed using the ICARE (Internal Dose Calculation for Radionuclide Exposure) software [23]. ICARE was developed as a tool for the calculation of reference and specific dose coefficients, retention and excretion functions for internal exposure to radionuclides. The current commercially available version of ICARE, which implements all biokinetic and dosimetric models used for the calculation of dose coefficients in ICRP Publication 119 [24], was updated as part of this study to include the most recent ICRP models and thus the specific dose coefficients calculated here are directly comparable with the reference dose coefficients of ICRP Publication 134 [9].

## 3. Results

### 3.1. In Vitro Tritium Release Study

**Figure 2:**
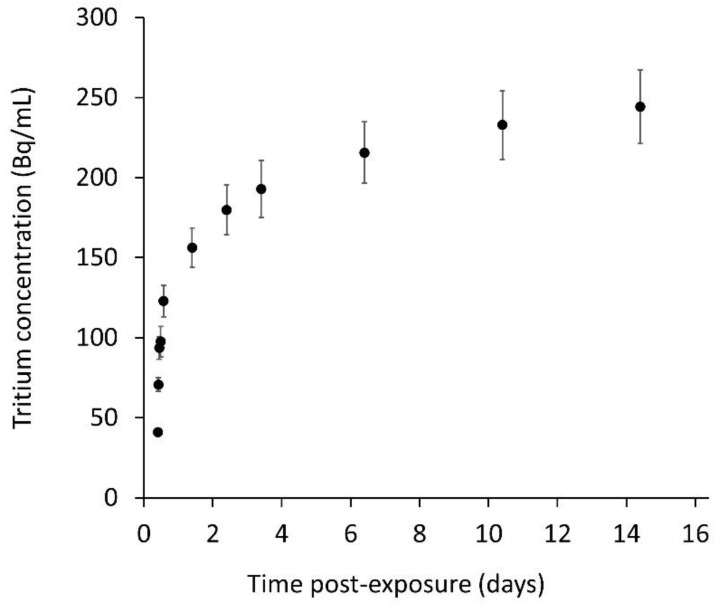
Release of tritium from tritiated steel particles: tritium concentration (Bq/mL) of lung lining fluid simulant (LLFS) samples.

The concentrations of tritium in the LLFS samples and derived fractional retention and release rates are indicated in Figure 2 and Figure S1. These show an initial fast release phase which slows significantly after day 1. The total tritium release over the duration of the 14-day study was 13% of the total, with 6% released within the first 4 h. This initial phase could perhaps represent release from a surface layer.

### 3.2. In Vivo Instillation Study

Measured muscle, liver and kidney sample masses and activities, and derived specific activities determined using the tissue digestion approach, are presented in the Supplementary Materials (Supplementary Data.xls). The average specific activities over animal groups (*n* = 4) are presented in Table 1 and Figure S2. The limits of detection (LOD) per sample were approximately 0.15 Bq (broadly equivalent to 0.5–1.5 Bq/g).

The results indicate some variability, especially for the kidney samples. The general trend for each tissue type is for an increase in specific activity to a maximum at 3 days post-exposure, which then reduces with time until 85 days, by which all results are below the limit of detection. From 7 days, the muscle- and liver-specific activities are very similar, but at earlier time points, the liver levels are higher by up to a factor of 60%. The kidney-specific activities are higher than the muscle and liver values by a factor of between 2 and 5 to 7 days and greater beyond, to a maximum factor of 20. This suggests possibly slower clearance in kidney, but, as noted previously, the variability in the kidney values is significantly higher than in the liver and muscle values, which makes it difficult to draw firm conclusions. The total available tritium in the liver samples for the day 1, 3 and 7 groups were also determined using the pyrolysis method. The average specific activity was 90 ± 20 Bq/g (mean ± SD) at 1 day, 115 ± 45 Bq/g at 3 days and 49 ± 8 Bq/g at 7 days. The temporal pattern of activity is the same as that seen in the results using the digestion approach, i.e., an increase from 1 to 3 days, followed by a reduction. However, the results using the digestion approach are, on average, 40–65% higher, although again, there was significant variability within groups. For each animal, two lung samples were analysed for total available tritium using the digestion approach: right top lung lobe and right middle lung lobe. The sample masses, activities and derived specific activities for each lobe are presented in the Supplementary Materials (Supplementary Data.xls). Average specific activities for the lung are presented in Table 1 and Figure S3. These results indicate, in general terms, a reduction in specific activity over time; however, there is very significant variability between animals. The pattern is similar for each lung lobe; however, the specific activity of the right top lobe is greater than that of the right middle lobe by a factor of between 2 and 40. For both lobes, the activity concentrations at the final time point, 85 d, were below the limit of detection.

**Figure 3:**
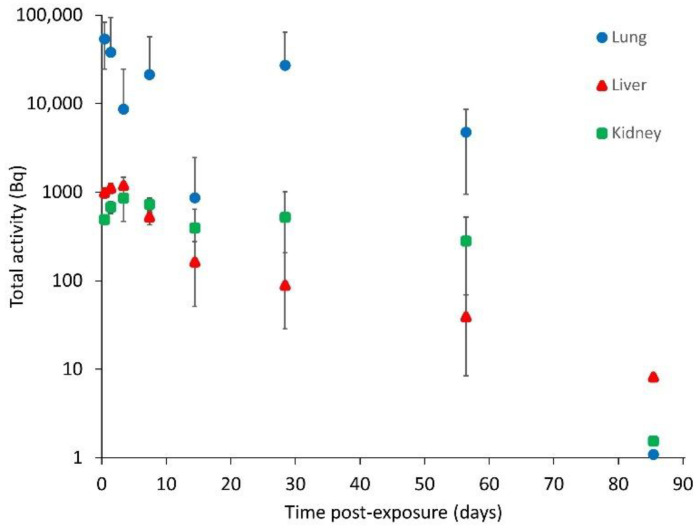
Tritium in lung (blue circle), liver (red triangle) and kidney (green square) (mean ± SD) (note that values without error bars at 85 days are at limit of detection and are included for indicative purposes only).

The total activity in liver, kidney and lung at each time point (Table 2 and Figure 3) was estimated by multiplying the specific activities by organ masses at the different time points determined by extrapolating the data on organ and tissue masses at different ages from Piao et al. [25] (Table S4).

The results for lung indicate a total activity of approximately 54 kBq at 1 h post-exposure, which is significantly less (by approximately a factor of 20) than the 1 MBq target instillation value. This is not unsurprising, as much of the tritium may remain bound within the particle matrix and the measurement of the total available tritium activity does not include this component. At all timepoints, the activity in the lung is higher than that in the liver or kidney (factors of between 2 and 300).

An approximate estimate was also made of the total tritium in all rat tissues aside from the lung using the specific activity measurements for muscle. For this purpose, it was assumed that all soft tissue and adipose tissue contained tritium at the same activity concentration as muscle tissue (i.e., skeletal tissue was excluded). Using µCT whole body composition measurements, Granton et al. [26] determined that for female Sprague Dawley rats, 22 ± 7% of the total body volume is adipose tissue, 72 ± 6% lean tissue and 6 ± 1% skeletal tissue and, further, using density values for the different tissue types of, respectively, 0.95, 1.05 and 1.92 gcm^−3^ [27], estimated mass percentages of 20 ± 6%, 70 ± 6% and 10 ± 4%, respectively. The estimated total soft tissue tritium activity is presented in Table 2. The estimated total soft tissue tritium activity (non-lung) reaches a peak at day 3 of 37 kBq and then reduces to 18 kBq at 7 days. The total soft tissue activity (non-lung) at days 14, 28 and 56 are similar, with the value at 56 days (6 kBq) slightly higher than those at 14 (5.5 kBq) and 28 (4 kBq) days, suggesting some variability between groups. The total activity varies from an initial peak of 75 kBq with a general downward trend to 11 kBq at 56 days, with the exception of the day 14 value, again reflecting the significant variability in the lung activities discussed earlier.

**Figure 4:**
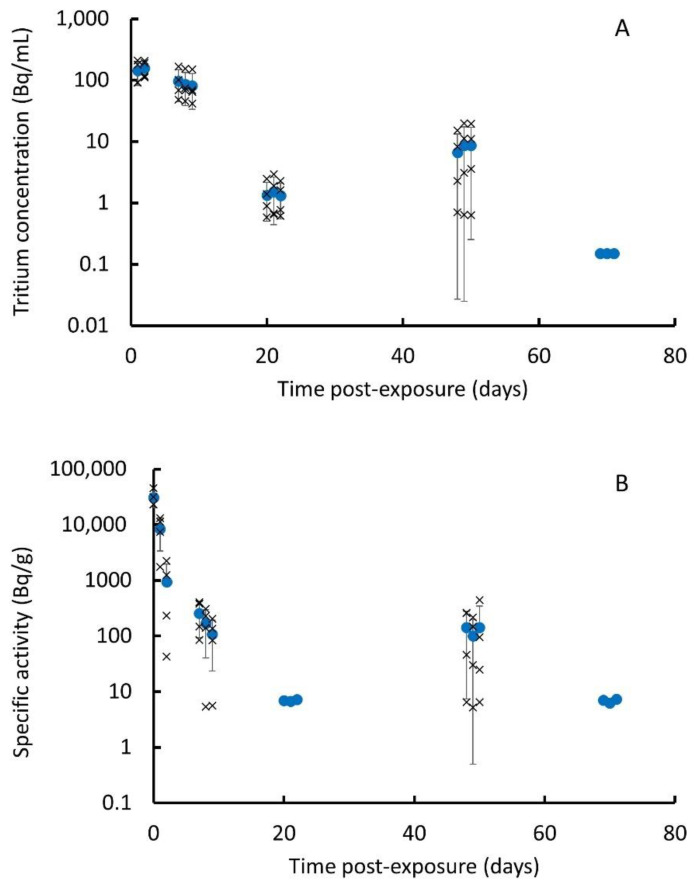
Tritium excretion: (**A**) tritium concentration of urine samples; and (**B**) specific activity of faecal samples (crosses are values for single animals, solid circles and error bars are mean (*n* = 4) ± SD. Note that values without error bars are at limit of detection and are included for indicative purposes only).

The measured daily urine sample tritium concentrations and daily group averages (*n* = 4) are presented in the Supplementary Materials (Supplementary Data.xls) and Figure 4A. These indicate average levels of 143 Bq/mL at day 1, rising to 157 Bq/mL at day 2 and then generally reducing until below the limit of detection (0.15 Bq/mL) by day 69, although the values on days 48–50 are higher than those on days 20–22, showing significant variability. Although there is variability between the animals at each time point, the day-to-day variation for individual animals is small. The measured faecal sample masses and activities and derived specific activities are presented in the Supplementary Materials (Supplementary Data.xls) with group averages in Figure 4B. These indicate specific activities peaking at 31 kBq/g at 1 h and then reducing rapidly with a reduction of over a factor of 100 by day 7. All samples for days 69 to 71 were below the limits of detection (5–7.5 Bq/g). The average specific activities for days 48–50 ranged from 100 to 140 Bq/g, although a limited number of samples were below the limits of detection, indicating a wide variability. Surprisingly, all specific activities for days 20 to 22 were also below the limits of detection, indicating some variability between the animal groups.

A number of daily urine production volumes were measured, generating an average production of 30.1 ± 6.9 mL/d (*n* = 3). Although a limited number of samples were taken, this is consistent with the values for female rats in the literature (e.g., 85 ± 8.7 mL/d/kg [28]). The daily tritium excretion rates are presented in Figure S4. The total daily excreted mass measurements and derived total daily excreted activities are presented in the Supplementary Materials (Supplementary Data.xls) and Figure S4. A comparison of excretion by faeces and urine (Figure S4) indicates that this is dominated initially by faeces, but after day 2, the tritium excretion by urine is greater or broadly similar to that by faeces. OBT and HTO were determined in 1 d and 7 d liver samples. The results (Table S5) indicate that the majority of the tritium (>97%) is OBT.

### 3.3. Biokinetic Model of Rat

**Figure 5:**
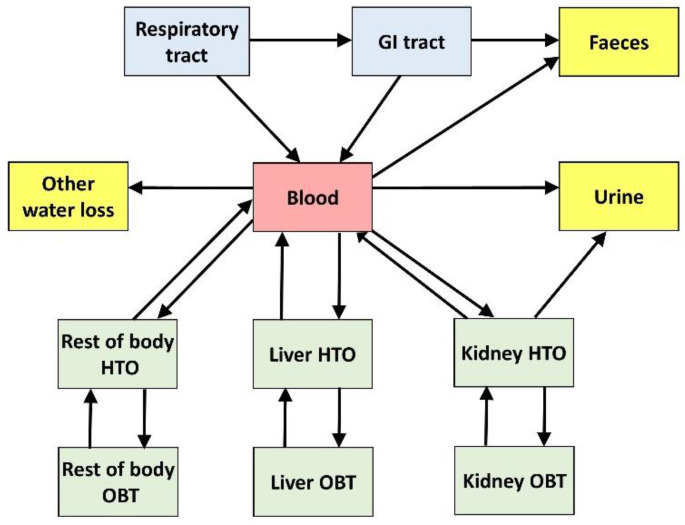
Structure of the rat biokinetic model.

The biokinetic model developed is shown in Figure 5. To represent the deposition and clearance of tritiated steel particles in the rat airways, the ICRP rat respiratory tract model was used [29]. In this model, it is assumed that, after intratracheal instillation, 83% of the administered activity deposits in the alveolar–interstitial (AI) region and 17% deposits in the tracheobronchial (TB) region. Mechanical (mucociliary) clearance from the AI region is represented by two compartments: AI1 (receiving 66% of initial alveolar deposit and cleared at a rate *m*_f_ = 0.0228 d^−1^) and AI2 (receiving 34% of initial alveolar deposit and cleared at a rate *m*_s_ = 0.0028 d^−1^). Mechanical clearance from the TB region transports activity to the extrathoracic (ET) region and then to the gastrointestinal (GI) tract. The release of activity from the particles and their subsequent transport to blood is represented by a rapidly released fraction, *f*_r_, of lung activity cleared at a rapid rate, *s*_r_, while the remainder, (1—*f*_r_), is cleared at a slow rate, *s*_s_. A fraction, *f*_b_, of the released activity may bind to lung tissues and be released to blood at a rate, *s*_b_; the rest of the activity is considered to be immediately transferred to blood. In the absence of direct evidence for a bound fraction, *f*_b_ is assumed to be zero.

The rat GI tract was simply represented by a single compartment, with the transfer rate to faeces determined by fitting to experimental excretion data. The tritium released from steel particles and transferred to blood is assumed to be in the form of HTO, which mixes rapidly with whole-body water after its entry into blood. A portion of tritium reaching blood as HTO becomes organically bound to carbon atoms due to biosynthesis in the body. The resulting OBT is generally non-exchangeable with hydrogen in body water and has a lower rate of turnover than HTO. The organic binding of tritium reaching blood as HTO and the turnover time of OBT in a given tissue depend on the tissue’s metabolic activity. Former measurements on laboratory animals indicate that 1–5% of HTO entering blood becomes incorporated into the organic components of tissues [9]. Here, the systemic model describing the distribution of tritium between blood, organs and tissues as well as its clearance to excreta was adapted to the available experimental data. The systemic body compartments considered were blood, liver, kidney and the rest of the body (RoB). For comparison with the experimental data, the activity retained in RoB was extrapolated from that measured in muscle. A fraction of HTO activity in each of these systemic compartments is exchanged with a companion compartment representing the OBT content of the organ. The excretion of HTO mainly takes place in urine; however, a significant portion of it is also cleared by the exhalation of water vapor and loss through skin (sweat plus insensible loss) and a smaller fraction is excreted in faeces [30]. The systemic and GI tract (GIT) transfer rates for the model are indicated in Table 3. These were determined by fitting the experimental data to the model. The fits obtained with SAAM II by minimising the deviation of the model to the activity data (points) are illustrated in Figure S5. The adjustment of the model to the data looks reasonable, taking into account the dispersion of the data.

**Figure 6:**
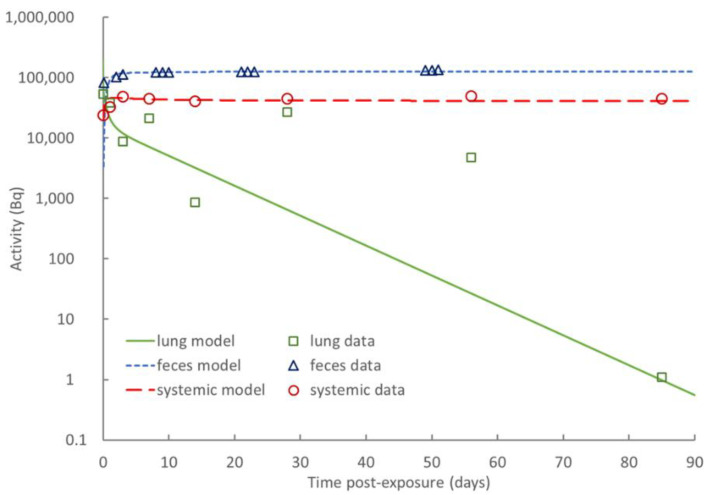
Fit of rat biokinetic model to experimental data.

Best estimates of absorption parameters from lung to blood were also obtained from the experimental data by comparing the kinetics of lung retention with that of systemic activity (retention in liver, kidney and RoB) plus cumulative excretion (see Figure 6).

The best estimates of the absorption parameter values are indicated in Table 4. In the absence of particle transport, these biokinetic parameters would predict 10% of the initial lung deposit retention in lung at 30 d and nearly complete clearance at 180 d post-instillation. According to ICRP [29], this indicates absorption borderline between fast (Type F) and moderate (Type M). The release of tritium from tritiated steel particles in the lungs therefore appears slightly faster than for tritiated glass fragments, luminous paint, titanium tritide and zirconium tritide that are assigned to Type M by ICRP [9]. However, the absorption through the gut appears to be relatively low.

### 3.4. Dose Coefficients for Workers Exposed to Tritiated Steel Particles

The specific committed effective dose coefficient calculated for the inhalation of tritiated steel particles by workers is 5.6 × 10^−12^ Sv Bq^−1^. The equivalent dose to the lung is 1.8 × 10^−11^ Sv Bq^−1^ for the male and 2.2 × 10^−11^ Sv Bq^−1^ for the female, while the equivalent dose to the systemic tissues is 3.0 × 10^−12^ Sv Bq^−1^ for the male and 3.6 × 10^−12^ Sv Bq^−1^ for the female. These values of dose per unit intake were obtained assuming the ICRP default activity median aerodynamic diameter (AMAD) for workers of 5 µm [21]. Equivalent calculations were undertaken assuming an AMAD of 13.3 µm, consistent with the particles used in the biokinetic study and also typical of the size of particles produced from the saw cutting of steel [13], as an example of further adaptation of dose coefficients to specific exposure situations. The corresponding committed effective dose coefficient is 3.3 × 10^−12^ Sv Bq^−1^. The equivalent dose to the lung is 6.7 × 10^−12^ Sv Bq^−1^ for the male and 8.2 × 10^−12^ Sv Bq^−1^ for the female, while the equivalent dose to the systemic tissues is 2.2 × 10^−12^ Sv Bq^−1^ for the male and 2.7 × 10^−12^ Sv Bq^−1^ for the female.

## 4. Discussion

The in vitro study measured the release of tritium from tritiated steel particles into a lung lining fluid simulant over 14 days. This found the limited release of tritium from the tritiated steel particles during the experimental period, with an initial fast release phase followed by a significantly slower release rate. In total, 13% was released, with 6% within the first 4 h. Information on tritium release can be used as an input to modelling studies on the biokinetics of tritium from inhaled particles. However, as discussed earlier, this is expected to be most relevant in the early stage following inhalation. The results of similar studies undertaken with metal tritides in biological fluid simulants indicate a wide range of release rates (Table S6). For example, hafnium tritide had <% tritium release after 200 days [31,32] whilst for titanium tritide, over 99% of the tritium was released by 200 days [33]. The result for zirconium tritide was between these two with 30% cleared by 200 days [12]. The fractional release rates in this study were greater than for hafnium tritide and varied from approximately 10^−1^ d^−1^ at day 1 to 10^−3^ d^−1^ at day 14 (Figure 2). This is similar to that for zirconium and titanium tritide during the first few weeks, but the trend is more similar to zirconium tritide over the longer term [34].

However, it is important to note that there are various factors that can influence release rates, including particle size, and it is possible that some of the differences between the hafnium results and those for titanium and zirconium relate to the larger size of the hafnium particles. For example, Cheng et al. [33] compared tritium release rates from titanium tritide of two particles sizes, 0.95 µm and 103 µm (count median diameter, CMD), and found significantly faster tritium release for the smaller particles, with fractional release rates varying from 10^−1^ d^−1^ at day 1 to 10^−2^ d^−1^ at day 30 for the smaller particles and an order of magnitude lower for the larger particles. The results for the tritiated steel particles used here (3 µm (CMD)) were between those for the two different sized titanium tritide particles. Tritium release from other forms of tritiated materials has also been investigated using a similar approach. For example, Cool and Maillie [35] found a fast release of tritium from tritiated glass particles. A number of different carbonaceous materials of similar particle size have also been investigated, with studies finding a range of retention fractions from almost 100% to 75% over 100 days depending upon the source and detailed composition of the materials [18,36]. Interestingly, another study with different sized carbon particles found that the release was greater for larger than smaller particles [17], the reverse of the trend for the titanium tritide particles.

The in vivo instillation study followed female Sprague Dawley rats for 3 months following the intratracheal instillation of ca 1 MBq of tritiated steel particles, collecting organ, tissue and excreta samples at various post-exposure time points and analysing them for total available tritium and, for a subset of samples, OBT content. The sum of the estimated total available activity in the rat at 1 day and the activity excreted to 1 day (total available tritium) is approximately 15% of the instilled activity, indicating that the majority of the tritium remains bound to the particles. This is similar to, but greater than would be anticipated from the in vitro study, which found 8% release by day 1. Perhaps, as discussed earlier, this is a result of increased tritium release from particles in the GI tract and also following phagocytosis within the lung. However, the uncertainty surrounding the estimate of total soft tissue tritium must be acknowledged. This finding is contrary to a study of metal tritides that found in vitro tritium release rates were typically higher than those in vivo [34].

Although it addressed the main components necessary for biokinetic modelling, this study did not consider the release of tritium in exhaled breath, a known excretion pathway. However, the results from other rodent studies have found this pathway not to be significant [10,11,12]. A limiting factor to this study is the variability of the results, especially for the lung samples. This could be due to the relatively small size of the samples analysed (~200 mg) in comparison to the whole lung (~1 g) and reflect the heterogeneity often seen in relation to lung deposition following instillation. This is supported somewhat by the results for other organs and tissues, e.g., liver and muscle, and excreta, which generally show lower levels of variability and a clearer temporal pattern, especially at the earlier time points. Another concern is that the results for the final post-exposure time do not appear fully consistent with those for other groups as they are all below the limits of detection, which for some organs and tissues suggests a very rapid reduction that does not follow the pattern of previous time points (Figure 3). The group sacrificed at the final time point provided the excreta samples for the 20–23-day and 69–72-day collection periods, which also appear to have lower tritium levels than would be expected given the results from the other groups (Figure 4), although the difference is not as marked as for the tissues. This could suggest that the instillation procedure may have been less efficient for this group.

A number of similar studies using metal tritides have been undertaken and reported in the literature (Table S7); however, it is difficult to directly compare their results with those from this study, as different analytical techniques and assumptions were made. For example, two studies used an analysis technique for some samples that partially dissolved the particles (base digestion) and introduced a correction factor [10,11]. The second of these and the zirconium tritide study also used a combustion technique and analysed wet and dry samples to estimate HTO and OBT levels separately [11,12], making the assumption that all of the tritium in the dry samples was OBT. For these reasons, the following is only a limited analysis of the literature in the area in comparison to this study.

The tritium detected in the lung following instillation of the tritiated steel particles was 5% of the instilled activity at 1 h post-exposure, reducing to 0.4% at 56 days. This is lower than that seen in an in vivo study using titanium tritide, which found 8% of the instilled activity in the lung at 3 days, reducing to 3% at 61 days [10]. Conversely, the faecal excretion rates were initially higher in the steel study, 8% at 1 day, reducing to 0.04% by day 50, than in the titanium tritide study, where excretion reduced from 1% to 0.03% over a similar period. The pattern of tritium excretion by urine was the reverse, with levels falling from 0.43% to 0.026% for the steel over a comparable timescale to the titanium tritide study reducing from 4% to 0.04%. In the steel study, the activity concentration seen in liver, kidney and muscle initially increased to a peak at 3 days and then reduced over time. This pattern of an initial increase has been seen in other studies of tritiated particles, where for zirconium tritide [12] and hafnium tritide [11], the values increased to a peak value within 1 to 2 weeks followed by a reduction.

The measurements of laboratory animals reviewed by ICRP [9] indicate that 1–5% of HTO entering blood becomes incorporated into the organic components of tissues and that human data suggest a range of 0.5–3%. This is in sharp contrast to the findings of this study, which indicate that over 97% of the tritium in the liver is OBT at 1–7 days post-exposure. However, there are a number of different techniques used for determining OBT levels, all of which make assumptions about which component of the tritium detected originated in the form of OBT, which makes it very difficult to compare. For example, in our study using a combustion technique, the assumption was made that the tritium released when the sample temperature was above 180°C was OBT. This contrasts with the in vivo studies of zirconium and hafnium tritides, which considered wet and dry samples and assumed the tritium in the dry samples was all OBT, although the details of the techniques used for the drying stage are unclear [11,12]. In the zirconium tritide study, the majority of the available tritium (>90%) was estimated to be present as OBT in the lung, liver, kidney and muscle, consistent with our results. In a more recent rodent study of orally administered HTO, Lee et al. [37] found that the OBT in tissues was approximately 36% of the total tritium at day 7 and 65% at day 13. However, the method used was based on a freeze-drying vacuum system significantly different to that used here. Interlaboratory intercomparison exercises could usefully be undertaken in this area to develop consistent approaches and reduce the level of uncertainty.

A rat biokinetic model for the inhalation of tritiated steel particles was built upon the experimental data. The structure of this model is relatively close to that used by ICRP for the calculation of dose coefficients. However, it considers a more detailed distribution of tritium in liver and kidney, while all systemic organs are treated as a global pool in the current ICRP models for tritium. Despite the transfer rates derived for the rat not being directly transposable to humans, they could be considered in future revisions of the reference biokinetic models for the tritium used in radiation protection. Of special interest is the relatively high specific activity in kidney, which suggests that the renal excretion pathway should specifically be considered for tritiated water clearance. Specific values were derived from the biokinetic model for lung absorption parameters (Table 4). These specific absorption parameter values can be and were extrapolated to humans. In conjunction with the start-of-art dosimetric models of the ICRP, specific equivalent and effective dose coefficients were evaluated with the dedicated ICARE software. A specific committed effective dose coefficient of 5.6 × 10^−12^ Sv Bq^−1^ was assessed for the inhalation of tritiated steel particles that may be applied, for instance, to workers during dismantling operations. Such results may valuably be considered in future revisions of ICRP dose coefficients for tritium. The dose coefficient evaluated here is slightly lower than that formerly assigned to other tritiated compounds (2.4 × 10^−11^ Sv Bq^−1^) and is also lower than that for HTO (2.0 × 10^−11^ Sv Bq^−1^) [9]. Dose coefficients were evaluated here for the ICRP reference AMAD of 5 µm for aerosols inhaled by workers and also for the specific AMAD of 13.3 µm, which may be more representative of the actual aerosols generated during sawing operations of stainless-steel pieces. However, in considering their relevance to decommissioning activities, factors such as the impact of particle shape should be addressed. The model particles used here, although of a similar size, are spherical, whereas those generated from model decommissioning activities were much more irregular, including bent platelet forms [13]. This could result in differences in tritium release rates, especially if “real” particles have a greater surface area per unit mass. When future characterisations of airborne particle size distributions are conducted for specific dismantling or maintenance operations, the estimated AMAD can be used to recalculate an AMAD-specific dose coefficient. As new experimental results become available, the present work might be improved to take advantage of a better characterisation of the different chemical forms of tritium within organs, i.e., bound within steel particles and/or present as HTO and OBT.

## 5. Conclusions

The results from an in vivo study of the biokinetics of tritiated steel particles—to the authors’ knowledge, the first to be reported—have been combined with state-of-the-art modelling to derive dose coefficients for the inhalation of tritiated steel particles, thus improving the accuracy of internal dosimetry for these particles. The results will support appropriate dose and risk management procedures and processes for activities involving tritiated steel, including the decommissioning of fission and fusion plants.

## Figures and Tables

**Table 1:** Specific activity of muscle, liver, kidney and lung samples.

Post-Exposure Time (d)	Specific Activity (Bq/g) *			
	Muscle	Liver	Kidney	Lung
0.04	78 ± 7	128 ± 16	214 ± 14	34,880 ± 18,990
1	96 ± 9	144 ± 18	296 ± 46	24,660 ± 36,360
3	137 ± 20	153 ± 37	373 ± 170	5613 ± 10,322
7	68 ± 11	68 ± 12	316 ± 59	13,710 ± 22,810
14	20 ± 7	21 ± 14	170 ± 107	555 ± 1028
28	14 ± 10	11 ± 15	219 ± 207	17,290 ± 23,350
56	22 ± 14	4.9 ± 3.9	115 ± 100	3002 ± 2403
85	<LOD ^#^	<LOD	<LOD	<LOD

* Mean specific activity ± standard deviation (*n* = 4); ^#^ LOD, limit of detection.

**Table 2:** Tritium in lung, liver, kidney and total soft tissue.

Post-Exposure Time (d)	Total Activity (Bq) *			
	Lung	Liver	Kidney	Total Soft Tissue ^$^
0.04	53,890 ± 29,330	988 ± 124	491 ± 32	20,860 ± 1948
1	38,100 ± 56,180	1114 ± 138	680 ± 106	25,480 ± 2472
3	8701 ± 16,000	1188 ± 288	862 ± 392	36,700 ± 5412
7	21,250 ± 35,350	524 ± 95	729 ± 136	18,250 ± 2834
14	863 ± 1599	163 ± 112	395 ± 248	5524 ± 1840
28	27,150 ± 36,660	89 ± 117	520 ± 491	3857 ± 2674
56	4773 ± 3821	39 ± 31	281 ± 245	6087 ± 3987
85	<LOD ^#^	<LOD	<LOD	<LOD

* Mean activity ± standard deviation (*n* = 4); ^$^ Total soft tissue excluding lung; ^#^ LOD, limit of detection.

**Table 3:** Systemic transfer rates of the rat biokinetic model for inhalation of tritiated steel particles.

From	To	Transfer Coefficient (d^−1^)
Blood	Liver HTO	0.45
Blood	Kidney HTO	0.18
Blood	RoB * HTO	17
Blood	Urine	0.56
Blood	Faeces	0.04
Blood	Other water loss	0.41
Liver HTO	Blood	2.7
Kidney HTO	Blood	0.10
RoB HTO	Blood	4.0
Liver HTO	Liver OBT	0.025
Kidney HTO	Kidney OBT	0.01
RoB HTO	RoB OBT	0.03
Liver OBT	Liver HTO	0.10
Kidney OBT	Kidney HTO	0.20
RoB OBT	RoB HTO	0.05
Kidney HTO	Urine	1.0
GIT ^#^	Faeces	2.0
GIT	Blood	0.10

* RoB, rest of body; ^#^ GIT, gastrointestinal tract.

**Table 4:** Biokinetic model absorption parameter values.

Parameter	Value
Rapidly released fraction (f_r_)	0.30
Rapid release rate (s_r_)	100 d^−1^
Slow release rate (s_s_)	0.064 d^−1^
Absorbed fraction of activity entering GIT (f_A_)	0.052

## Data Availability

The data presented in this study are available from the authors on reasonable request.

## Supplementary Materials

The following supporting information can be downloaded at: https://www.mdpi.com/article/10.3390/toxics10100602/s1, 1. Supplementary Data.xls 2. Supplementary Tables and Figures including: Table S1: Gamble’s solution; Table S2: Samples collected; Table S3: Overview of operating cycle for pyrolysis approach; Table S4: Estimated organ masses; Table S5 Organically bound tritium (OBT) and tritiated water (HTO) in liver samples at 1 and 7 days post-exposure; Table S6: Studies in the literature on the release of tritium from tritiated particles; Table S7: Metal tritide biokinetic studies in the literature; Figure S1: Cumulative fraction of tritium released from (a) and retained in (b) the particles; Figure S2: Specific activity of muscle (a), liver (b) and kidney (c) samples; Figure S3: Specific activity of lung (*n* = 4) (circles) derived from values for top right lobe (squares) and right middle lobe (triangles); Figure S4: Daily excreted activity in faeces (purple squares) and urine (green diamonds) and total (x); Figure S5: Fitting of rat biokinetic model to experimental data: (a) lung; (b) kidney; (c) liver; (d) RoB; (f) urine; and (g) faeces.
