# Supplementary material for: Biokinetics and Internal Dosimetry of Tritiated Steel Particles
Source: Toxics. 2022 Oct 12;10(10):602. doi: 10.3390/toxics10100602 (PMC9607624; doi:10.3390/toxics10100602)
Supplement: Supplementary file 1 [file toxics-10-00602-s001.zip › Supplementary Information Tables and Figures V2.pdf]

Supplementary Information

Biokinetics and Internal Dosimetry of Tritiated Steel Particles

Rachel Smith [1,*], Michele Ellender [1], Chang Guo [1], Derek Hammond [1], Adam Laycock [1], Martin O. Leonard [1], Matthew Wright [1], Michael Davidson [1], Véronique Malard [2], Mickaël Payet [3], Christian Grisolia [3] and Eric Blanchardon [4,*]

[1] Radiation Chemicals and Environmental Hazards, UK Health Security Agency, Harwell Campus, Didcot OX11 0RQ, UK
[2] Biosciences and Biotechnology Institute of Aix-Marseille (BIAM) (Aix-Marseille University, French Alternative Energies and Atomic Energy Commission (CEA), French National Centre for Scientific Research (CNRS)), 13108 Saint Paul-Lez-Durance, France
[3] Institute for Magnetic Fusion Research (IRFM), French Alternative Energies and Atomic Energy Commission (CEA), 13108 Saint-Paul-lez-Durance, France
[4] Institut de Radioprotection et de Sûreté Nucléaire (IRSN), 92260 Fontenay-aux-Roses, France
* Correspondence: rachel.smith@ukhsa.gov.uk (R.S.); eric.blanchardon@irsn.fr (E.B.)

1. Supplementary Tables

Table S1. Gamble's solution*

Chemical	Composition	Concentration (g L^{-1})
Magnesium chloride	$MgCl_2$	0.095
Sodium chloride	$NaCl$	6.019
Potassium chloride	KCl	0.298
Sodium phosphate	Na_2HPO_4	0.126
Sodium sulphate	Na_2SO_4	0.063
Calcium chloride dihydride	$CaCl_2.2H_2O$	0.368
Sodium acetate	$C_2H_3O_2Na$	0.547
Sodium bicarbonate	$NaHCO_3$	2.604
Trisodium citrate dihydrate	$C_6H_5Na_3O7.2H_2O$	0.097

*From [1]

Table S2. Samples collected*

Sample name	Sample type	Sample description
BLD	Blood	Blood
L1	Lung	Right top lobe
L2	Lung	Right middle lobe
LTT	Lung	Left Lobe
Lv	Liver	Small sample from liver
LvTT	Liver	Sample from liver
Kid	Kidney	Small sample from kidney (~ 1/3 kidney)
M	Muscle	Small sample from left leg
MTT	Muscle	Larger muscle sample

*note not all samples were analysed

Table S3. Overview of operating cycle for pyrolysis approach

**Analysis of samples for total tritium**	**Analysis of samples for OBT and HTO separately**
Set up pyrolyser, start air flowing at ≈0.25 litres/minute	
Ramp temperature at 3°C/minute to 180°C	
Hold temperature at 180°C for 30 minutes	Hold temperature at 180°C for 90 minutes
No bubbler change required	Change bubbler for OBT collection 5 to 15 minutes before second ramp
Ramp temperature at 5°C/minute to 300°C	
Hold temperature at 300°C for 15 minutes	
Ramp temperature at 5°C/minute to 500°C	
Change gas to oxygen flowing at ≈0.25 litres/minute	
Ramp temperature at 5°C/minute to 900°C and hold for at least 30 minutes	
Turn off furnaces, open vents and turn on cooling fan	

Table S4. Estimated organ masses*

**Post-exposure time**	**Lung (g)**	**Liver (g)**	**Kidney (g)**	**Whole body (g)**	**RoB$ (g)**
1h	1.55	7.74	2.30	296.46	284.88
1d	1.55	7.74	2.30	296.46	284.88
3d	1.55	7.76	2.31	298.15	286.53
7d	1.55	7.76	2.31	298.15	286.53
14d	1.56	7.78	2.33	299.85	288.18
28d	1.57	7.85	2.37	304.92	293.13
56d	1.59	7.94	2.43	311.69	299.74
85d	1.61	8.02	2.49	318.46	306.34

*extrapolated from data in [2]

$ Rest of Body (RoB) mass estimated by subtracting lung, liver and kidney from whole body

Table S5. Organically bound tritium (OBT) and tritiated water (HTO) in liver samples at 1- and 7-days post-exposure

**sample no.**	**HTO Specific activity (Bq/g)**	**SD* (Bq/g)**	**OBT Specific activity (Bq/g)**	**SD (Bq/g)**	**Total Specific activity (Bq/g)**	**% OBT**
1 – 1d	0.59	0.14	106.00	24.00	106.59	99.5%
2 – 1d	0.04	0.03	61.00	14.00	61.04	99.9%
3 – 1d	0.48	0.12	92.00	21.00	92.48	99.5%
4 – 1d	0.19	0.05	100.00	23.00	100.19	99.8%
Average	0.33		89.75		90.08	99.7%
1 – 7d	0.24	0.07	53.00	12.00	53.24	99.6%
2 – 7d	0.50	0.12	45.00	10.00	45.50	98.9%
3 – 7d	1.50	0.38	56.00	13.00	57.60	97.2%
4 – 7d	< 0.03	NA	40.00	9.00	< 40.03	> 99.9%
Average$	0.59		48.50		49.09	98.8%

*Standard deviation
$Assuming 0.03 for sample 4

Table S6. Studies in the literature on the release of tritium from tritiated particles

Reference	Tritiated Material	Lung fluid simulant	Particle size µm (σg)	Results Release Rate (t/2)
Cool and Maillie (1983) [3]	Glass	Simulated lung fluid	3.8	98% half-time 13 d and 2% half-time 220 d
Cheng et al (1997) [4]	Titanium	SUF - serum ultrafiltrate	0.95<br>103	24% half-time 1 d and 76% half-time 33 d<br>Half-time 361 d
Inkret et al (2001) [5]	Hafnium	Simulated lung fluid (no details)	1	Study 1: 0.2% half-time 43d and 99.8% half-time @ $5x10^5$d<br>Study 2: approximately 0.05% release in 200 days
Cheng et al (2002) [6]	Hafnium	Simulated lung fluid	1.74	Very insoluble. Less than 1% release after 215 days<br>99.92% half-time $4.28x10^5$ d and 0.08% half-time 46 days
Hodgson et al (2002) [7]	Graphite	Lung serum simulant	Fine<br>Coarse	0.3% half-time 2 m and 99.7% half-time 1925 d<br>5% half-time 2 m and 95% half-time 110 d
Zhou and Cheng (2004) [8]	Hafnium<br>Titanium<br>Zirconium	SUF - serum ultrafiltrate	29.7 (2.41)<br>6.15 (1.93)<br>4.43 (2.24)	99.9237% half-time $4.28x10^5$ d and 0.076% half-time 46 d<br>24% half-time 1 d and 76% half-time 33 d<br>47.5% half-time 42 d and 52.5% half-time 385 d
Hodgson et al (2006) [9]	Graphite	Lung serum simulant	Not defined	For the most soluble materials<br>6.45% half-time 2 m, 8.69% half-time 1.3 d and 85% half-time 456 d
Zhou et al (2010) [10]	Zirconium	SUF - serum ultrafiltrate	0.34 (2.34)	Total tritium release 37% by 71 days<br>47.5% half-time 42 d and 52.5% half-time 385 d

Table S7. Metal tritide biokinetic studies in the literature

Material	Activity per animal	Particle Size	Animal type	Total animals	Time points (Grp no.)	Organs	Excreta	Exhaled air	Sample processing and counting	Ref
Titanium Tritide	1 MBq	CMD 0.95 µm GSD 1.93	F344/Crl rats (male) obtained t 8 weeks, 11-12 week at instillation	36	3, 14, 30, 61, 121 days (6 animals)	Lungs and bronchial lymph nodes	6 rats Individual met cages, urine and faeces collected daily for 10 days and then 5 consecutive days at 1, 2 and 4 months	Exhaled air from 2 of the metabolic cages collected at same times as excreta	Base digestion procedure. Counting corrected for dissolution of particles.	Cheng et al. 1999 [11]
Hafnium Tritide	1.55 MBq	CMD 1.04 µm GSD 2.41	F344/Crl rats (male) Obtained at 8 weeks age, not clear what age at instillation	52 (some confusion may be only 44)	1h, 1,2,3,7,14 days and 1,2,4,6 months (4 animals)	Lungs, bronchial lymph nodes, liver kidney, muscle, blood and GI tract	4 rats Individual met cages, urine and faeces collected daily for 14 days and then 3 consecutive days at 1,2,4 and 6 months	Exhaled air from 2 metabolism cages collected at same time as excreta	Sample analysis as for above study, except for urine which was counted directly and faeces which underwent a combustion technique. Indicates HTO and OBT separately measured.	Zhuo and Cheng 2003 [12]
Zirconium Tritide	0.925 MBq	CMD 0.34 µm GSD 2.24	F344/Crl rats (male) obtained at 7 weeks, 12-13 weeks at instillation	49	1h, 1,2,3,7, 14,30,60, 120,180 days (4 animals)	Lungs, bronchial lymph nodes, liver kidney, muscle, blood	4 rats Individual met cages, urine and faeces collected daily for 10 days and then 3 consecutive days at 1,2,4 and 6 months. Animals sacrificed at 180d	Exhaled air from 2 metabolism cages collected at same time as excreta	All tissue and excreta samples treated using a combustion method. Analysed in water part of tissue and dried (HTO, OBT).	Zhuo et al. 2010 [10]

2. Supplementary Figures

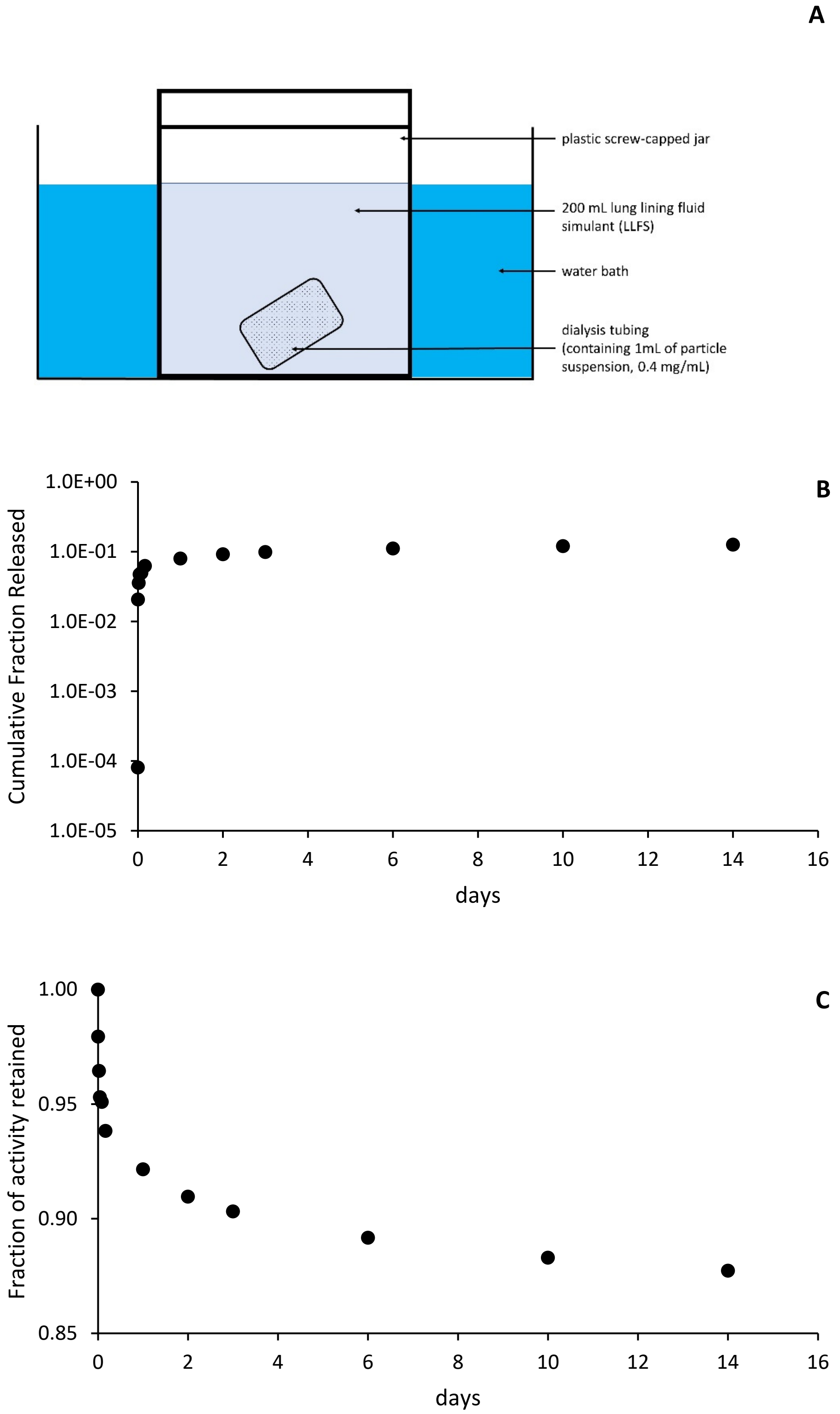

Figure S1. In vitro tritium release study. Schematic of experimental set-up (A) (note 1 mL samples taken from LLFS in screw-capped jar at times to 14 days and analysed for tritum content), cumulative fraction of tritium released from (B) and retained in (C) the particles.

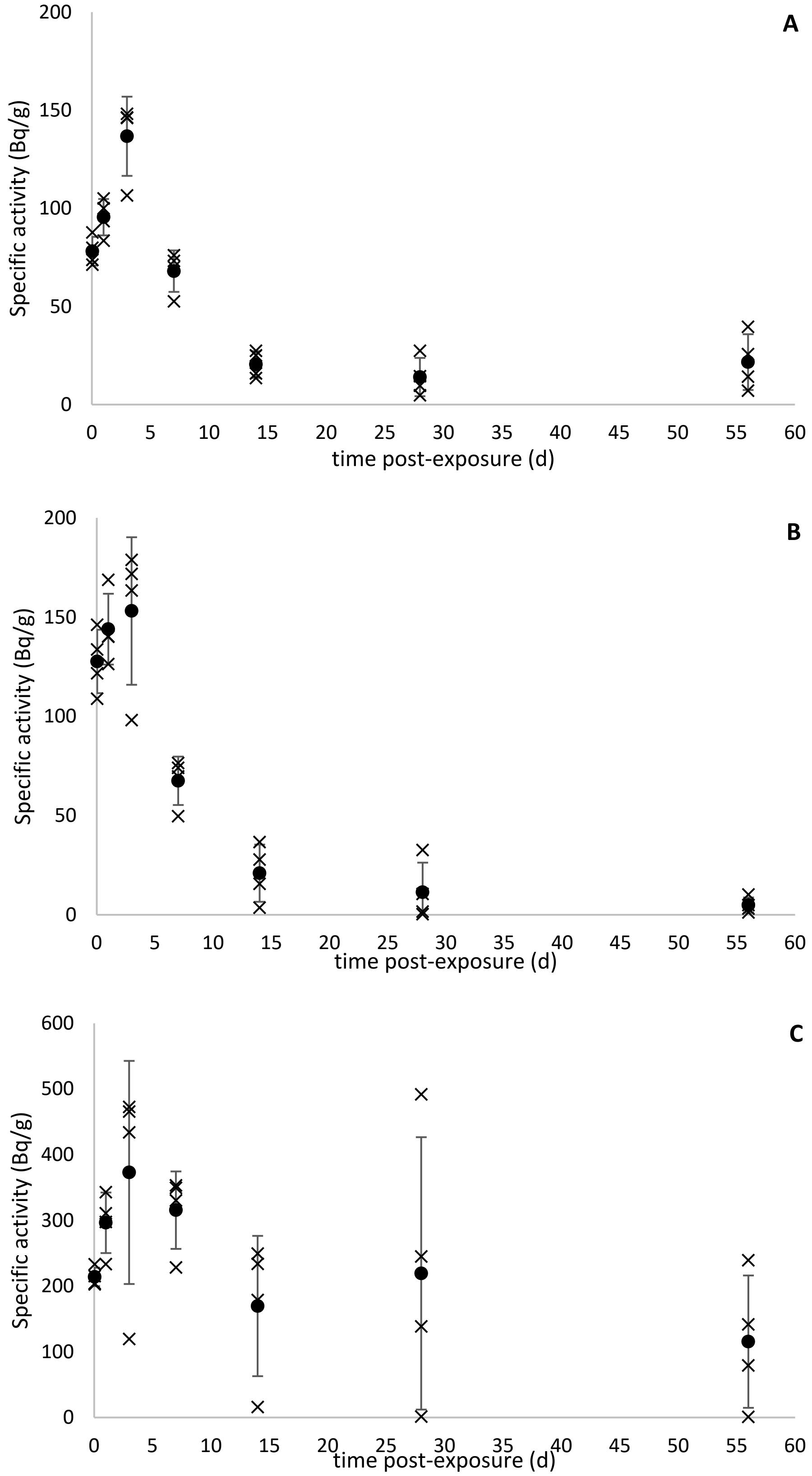

Figure S2. Specific activity of muscle (A), liver (B) and kidney (C) samples (mean ± SD).

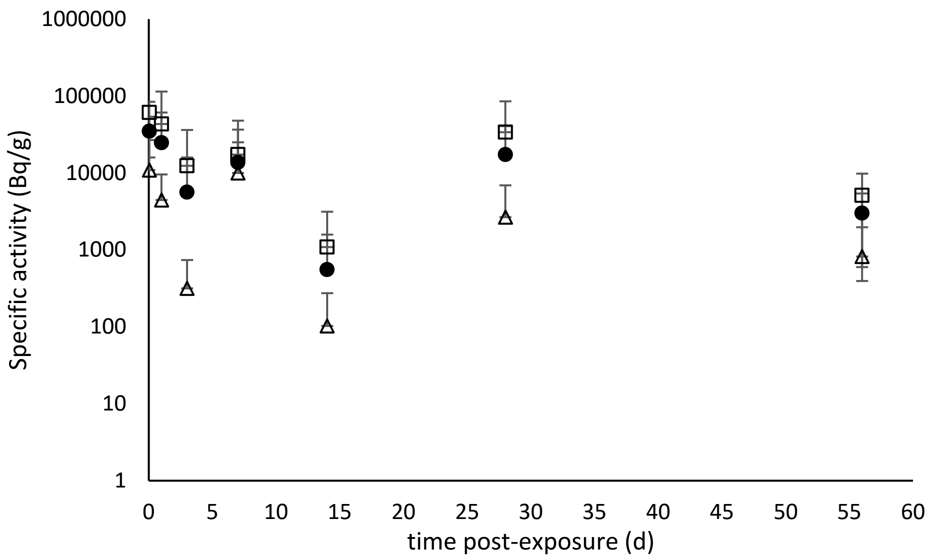

Figure S3. Specific activity of lung (n=4) (circles) derived from values for top right lobe (squares) and right middle lobe (triangles) (mean ± SD).

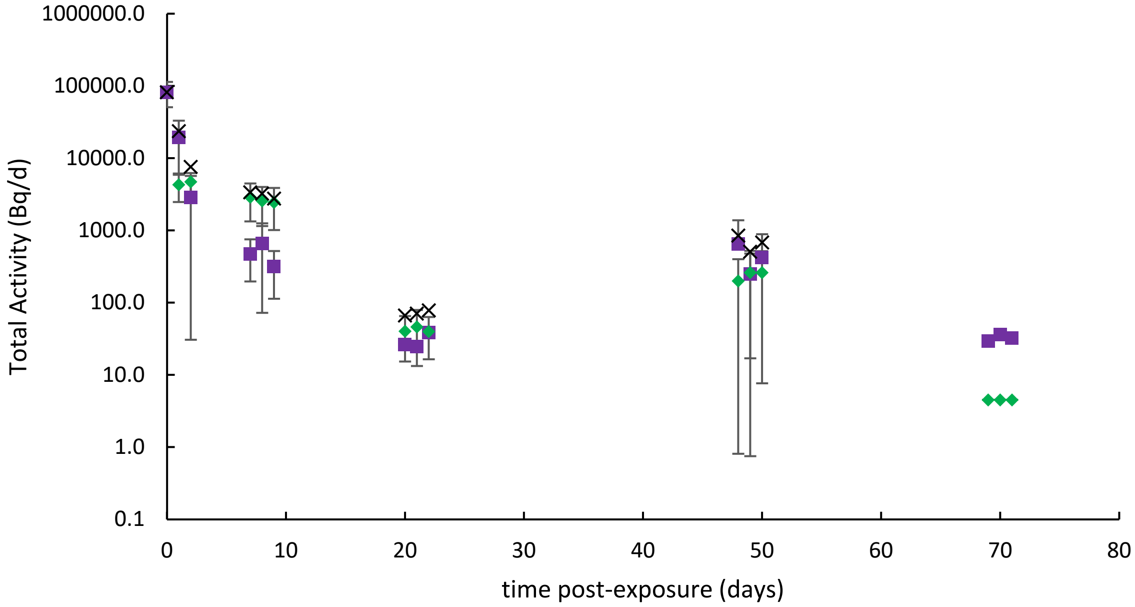

Figure S4. Daily excreted activity in faeces (purple squares) and urine (green diamonds) and total (x) (mean ± SD) (note that (i) value at d0 is for faeces only, and (ii) faeces and urine values without error bars are at limit of detection and are included for indicative purposes only).

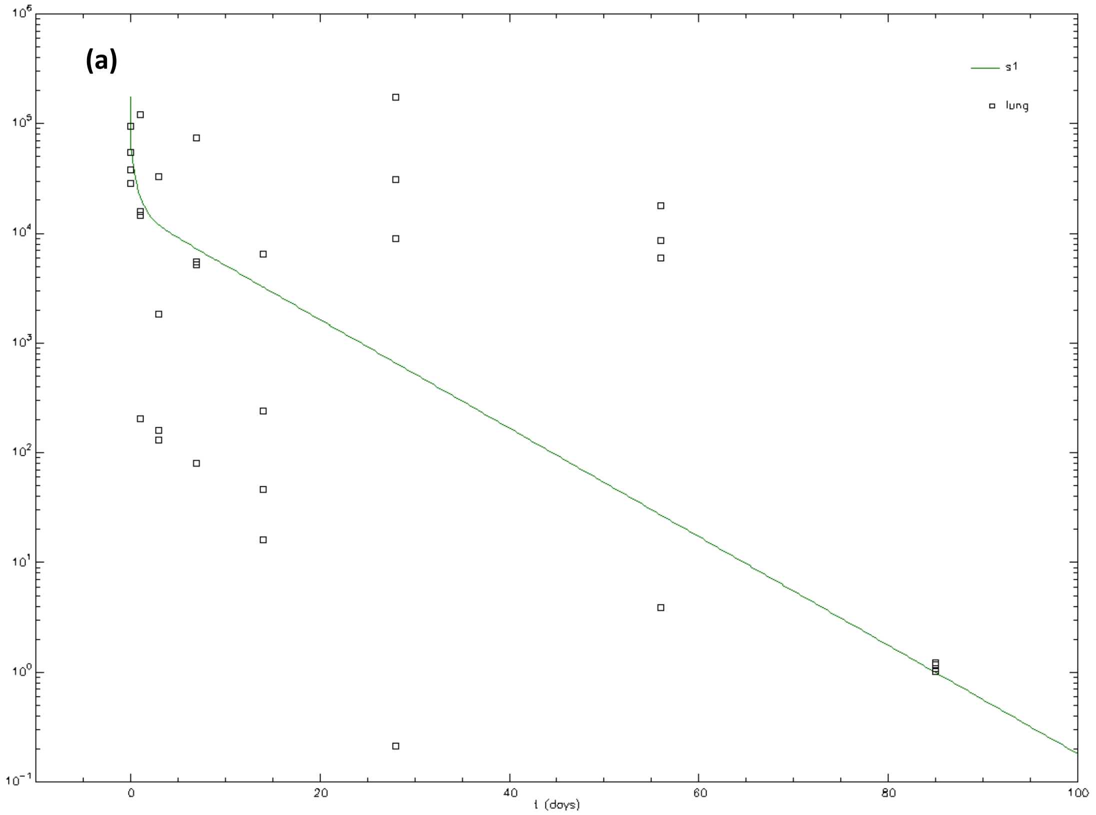
(a)
s1
lung
10^{6}
10^{5}
10^{4}
10^{3}
10^{2}
10^{1}
10^{0}
10^{-1}
0
20
40
60
80
100
t (days)

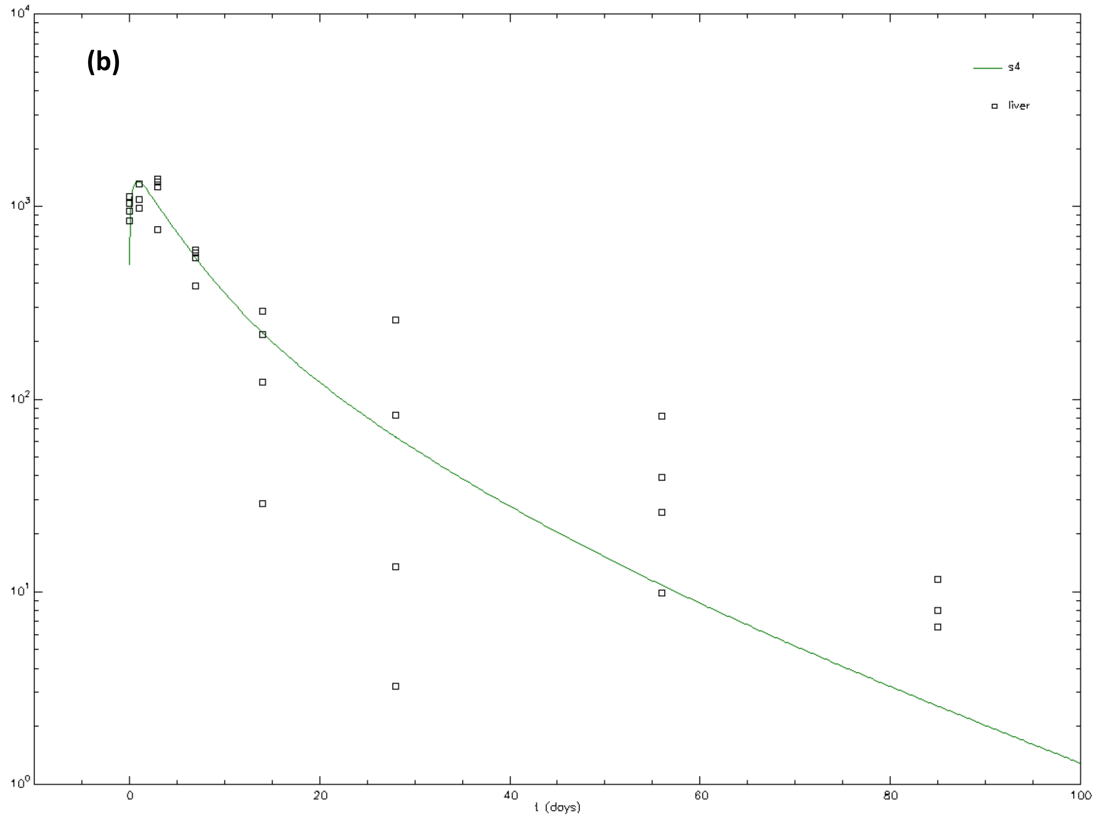
(b)
s4
liver
10^{4}
10^{3}
10^{2}
10^{1}
10^{0}
0
20
40
60
80
100
t (days)

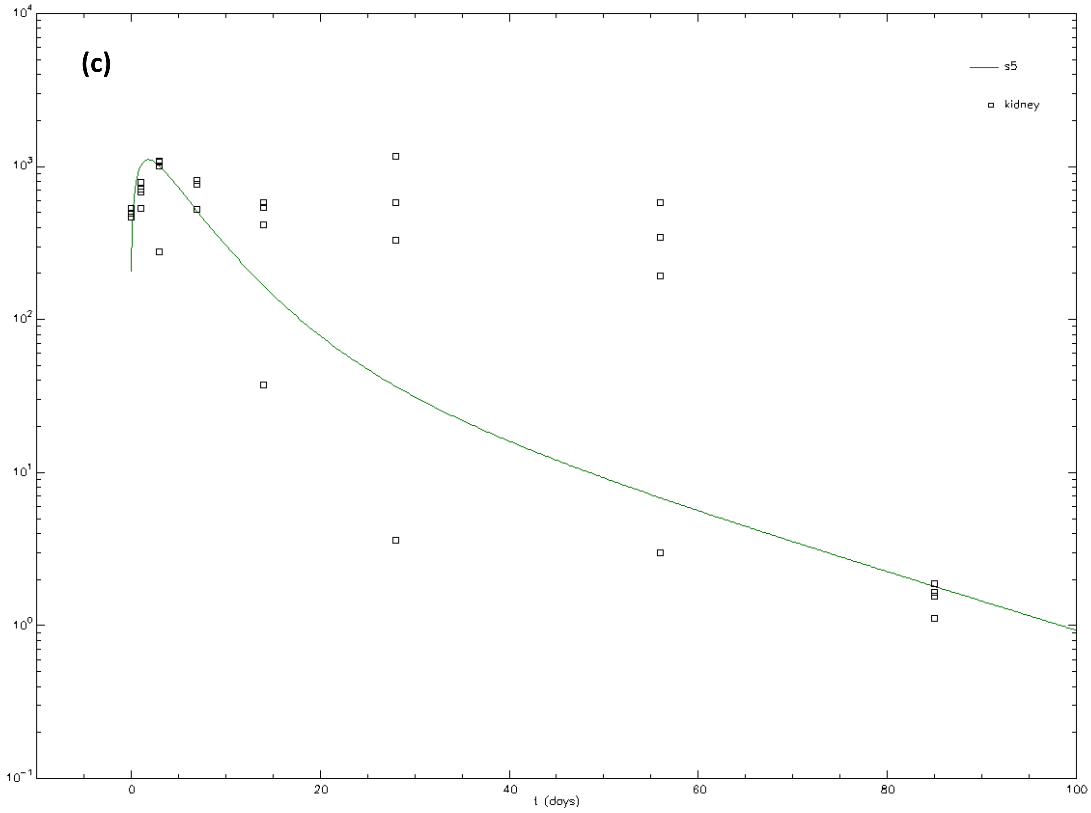
(c)
s5
kidney
t (days)

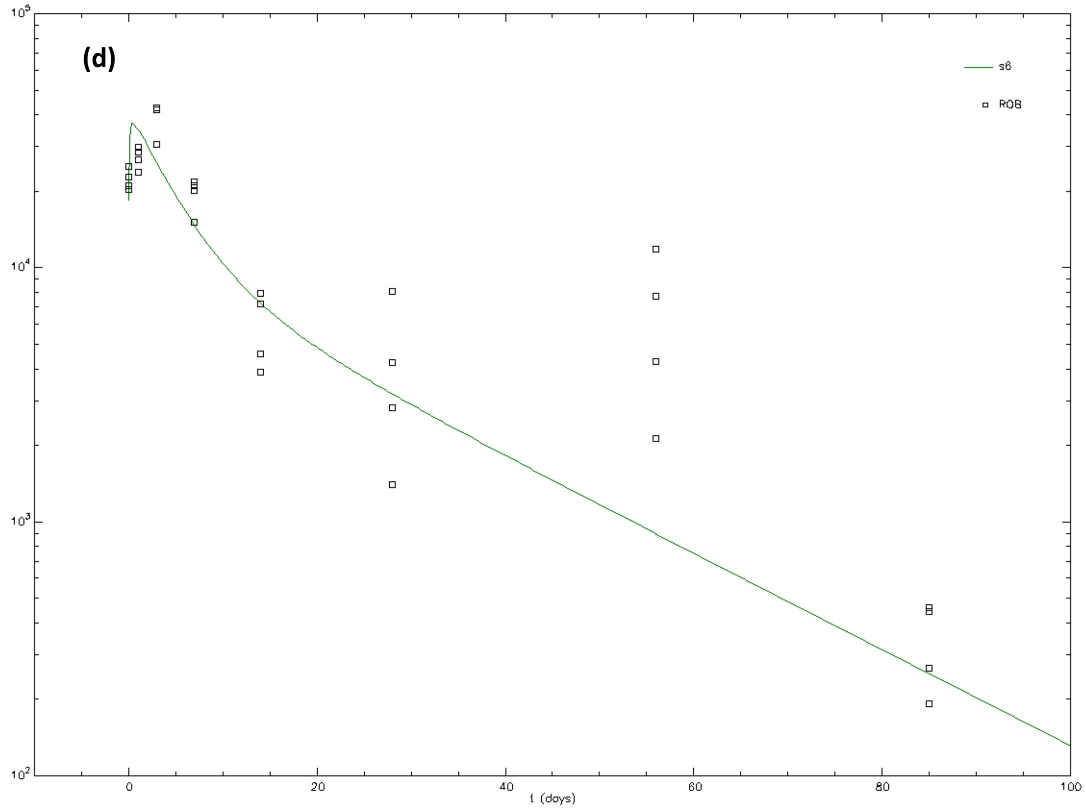
(d)
s6
ROB
t (days)

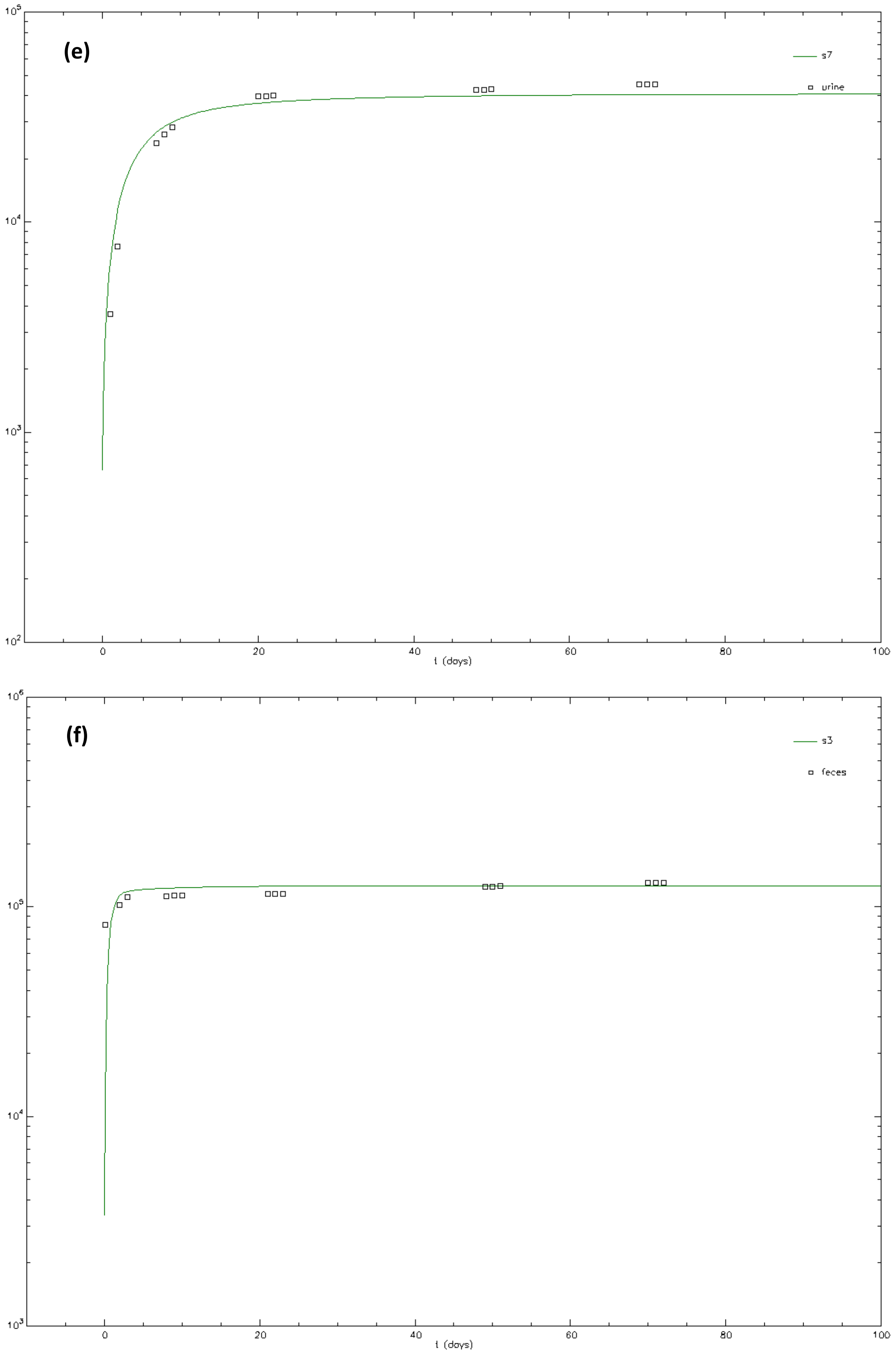

Figure S5. Fitting of rat biokinetic model to experimental data: (a) lung; (b) liver; (c) kidney; (d) rest of body (RoB); (e) urine; and (f) faeces. Tissue tritium content (Bq) in comparison to individual animals (n=4) and for excreta mean (n=4) cumulative excretion (Bq).

References

1. Liu, Y.; Qi, Y.; Yin, C.; Wang, S.; Zhang, S.; Xu, A.; Chen, W.; Liu, S. Bio-transformation of Graphene Oxide in Lung Fluids Significantly Enhances Its Photothermal Efficacy. *Nanotheranostics* **2018**, *2*, 222-232, doi:10.7150/ntno.25719.
2. Piao, Y.; Liu, Y.; Xie, X. Change trends of organ weight background data in sprague dawley rats at different ages. *Journal of toxicologic pathology* **2013**, *26*, 29-34, doi:10.1293/tox.26.29.
3. Cool, D.A.; Maillie, H.D. Dissolution of tritiated glass microballoon fragments: implications for inhalation exposure. *Health Phys* **1983**, *45*, 791-794.
4. Cheng, Y.-S.; Dahl, A.R.; Jow, H.N. Dissolution of Metal Tritides in a Simulated Lung Fluid. *Health Physics* **1997**, *73*.
5. Inkret, W.C.T.; Schillaci, M.E.; Boyce, M.K.; Cheng, Y.S.; Efurd, D.W.; Little, T.T.; Miller, G.; Musgrave, J.A.; Wermer, J.R. Internal Dosimetry for Inhalation of Hafnium Tritide Aerosols. *Radiation Protection Dosimetry* **2001**, *93*, 55-60, doi:10.1093/oxfordjournals.rpd.a006413.
6. Cheng, Y.-S.; Zhou, Y.; Wang, Y.-S.; Inkret, W.C.; Wermer, J.R. Dose Estimate of Inhaled Hafnium Tritide using the ICRP 66 Lung Model. *Health Physics* **2002**, *82*.
7. Hodgson, A.; Rance, E.R.; Pellow, P.G.D.; Stradling, G.N. *In vitro dissolution of tritium loaded carbon particles from the JET Tokamak*; NRPB-DA/1/2002, NRPB: Chilton, Oxfordshire, UK, 2002.
8. Zhou, Y.; Cheng, Y.S. Dosimetry of Metal Tritide Particles as Evaluated by the ICRP 66 Model and a Biokinetic Model from Laboratory Rats. *Health Physics* **2004**, *86*.
9. Hodgson, S.A.; Scott, J.E.; Hodgson, A. *In vitro dissolution of tritium loaded particles from the JET fusion machine*; RPD-DAR-02-2006, HPA: Chilton, Oxfordshire, UK, 2006.
10. Zhou, Y.; Cheng, Y.-S.; Wang, Y. Dissolution Rate and Biokinetic Model of Zirconium Tritide Particles in Rat Lungs. *Health Physics* **2010**, *98*.
11. Cheng, Y.-S.; Snipes, M.B.; Wang, Y.; Jow, H.-N. Biokinetics and Dosimetry of Titanium Tritide Particles in the Lung. *Health Physics* **1999**, *76*.
12. Zhou, Y.; Cheng, Y.S. Dose Assessment for Inhaling Hafnium Particles Based on Laboratory Rats Study. *Health Physics* **2003**, *84*.
